# Extra-Virgin Olive Oil in Alzheimer’s Disease: A Comprehensive Review of Cellular, Animal, and Clinical Studies

**DOI:** 10.3390/ijms25031914

**Published:** 2024-02-05

**Authors:** Amer E. Alkhalifa, Nour F. Al-Ghraiybah, Amal Kaddoumi

**Affiliations:** Department of Drug Discovery and Development, Harrison College of Pharmacy, Auburn University, 720 S Donahue Dr., Auburn, AL 36849, USA; aea0068@auburn.edu (A.E.A.); nfa0007@auburn.edu (N.F.A.-G.)

**Keywords:** Alzheimer’s disease (AD), olive oil, extra-virgin olive oil, EVOO, EVOO phenolic compounds, amyloid-β, tau, neuroinflammation, blood–brain barrier, autophagy, oxidative stress

## Abstract

Alzheimer’s disease (AD) is a progressive neurodegenerative disorder that is characterized by several pathological hallmarks, including the deposition of amyloid-β (Aβ) plaques, neurofibrillary tangles, blood–brain barrier (BBB) dysfunction, increased oxidative stress, and neuroinflammation. Current treatment options include monoclonal antibody drugs, acetylcholinesterase, and n-methyl-d-aspartate (NMDA) antagonists. Although those treatments provide some improvements in patients’ quality of life, they fail to prevent or cure AD. Current research aims to identify novel targets and tools for AD prevention and modification. In this context, several studies showed the beneficial effect of the Mediterranean diet in the prevention and treatment of AD. One integral component of the Mediterranean diet is olive oil and extra-virgin olive oil (EVOO), which is high in phenolic compounds. EVOO and other olive-related phenolic compounds have been shown to reduce the risk of developing mild cognitive impairment (MCI) and AD. In this review, we discuss the mechanisms by which EVOO and phenolic compounds exert neuroprotective effects, including modulation of AD pathologies and promotion of cognitive health. Findings indicate that EVOO and its phenolic constituents influence key pathological processes of AD, such as Aβ aggregation, tau phosphorylation, and neuroinflammation, while also enhancing BBB integrity and reducing oxidative stress. The human studies cited reveal a consistent trend where the consumption of olive oil is associated with cognitive benefits and a decreased risk of AD and related dementias. In conclusion, EVOO and its phenolic compounds hold promising potential for the prevention and treatment of AD, representing a significant shift towards more effective strategies against this complex neurodegenerative disorder.

## 1. Introduction

Alzheimer’s disease (AD) is a complex and progressive neurodegenerative disorder primarily affecting elderly people; it is characterized by cognitive decline, memory impairment, and impaired daily functioning [1]. AD is the leading cause of dementia, contributing to a substantial 60–80% of all dementia cases and ranking as the sixth leading cause of death among individuals aged 65 and above in the United States alone [2]. Aging represents the most formidable risk factor for AD, with a prevalence of 10% in individuals over 65 years old, which escalates to 40% in those over 80 years old [3]. As the global population continues to age, the frequency of AD is on the rise, rendering it a profound healthcare challenge. There are more than 55 million AD cases worldwide, which is projected to triple to 152 million by 2050. The economic implications are staggering, with AD expected to incur a cost of USD 1.1 trillion by 2050 [4]. Consequently, the surge in personal and financial burdens underscores the critical need for effective preclinical diagnosis and therapeutic management to prevent or arrest the disease’s progression before symptomatic onset.

AD is characterized by two primary neuropathological hallmarks: the buildup of amyloid-β (Aβ) plaques and the formation of neurofibrillary tangles (NFTs) in the brain [5]. Multiple hypotheses have been advanced to elucidate the development of AD, encompassing the amyloidogenic cascade, tauopathy, neurovascular dysfunction, oxidative stress, and neuroinflammation [6]. Notably, neurovascular dysfunction is increasingly recognized for its crucial role in AD’s onset and progression, linking cerebrovascular changes to neurodegenerative processes [7,8]. The blood–brain barrier (BBB) is essential for maintaining brain homeostasis and neuronal function, and its dysfunction in AD, characterized by heightened permeability, facilitates the entry of harmful substances and immune cells into the brain [7]. This exacerbates neuroinflammation and oxidative stress, accelerating AD pathology. BBB breakdown, preceding cognitive decline and Aβ accumulation, emerges as a potential early marker and therapeutic target in AD [8,9,10]. These pathological events induce neuronal atrophy and synaptic loss, culminating in neurodegeneration [5].

Despite extensive studies, the exact etiology of AD is not fully understood [11,12]. Many researchers agree that both genetic and environmental factors are involved, setting off a pathophysiological chain of events that eventually leads to AD pathology and dementia [13,14]. The most significant genetic risk factor for late-onset AD is the apolipoprotein E (APOE) gene, particularly the E4 allele, which is linked to increased risk and earlier onset of the disease [15,16]. Other genetic factors, including amyloid protein precursor (APP), presenilin 1 (PSEN1), and presenilin (PSEN2) gene mutations, are associated with early-onset AD [17,18]. Beyond genetics, lifestyle factors like diet, exercise, cognitive engagement, cardiovascular health, and environmental exposures also play pivotal roles in AD risk [19,20].

Recognizing the pressing need for therapeutic advancements, the U.S. Food and Drug Administration (FDA) has recently granted accelerated approval to Aducanumab and full approval to Lecanemab—novel treatments targeting Aβ, a key player in AD pathology [21,22]. These monoclonal antibody drugs represent significant strides in AD treatment. Yet the spectrum of available medications, such as acetylcholinesterase inhibitors (galantamine, rivastigmine, and donepezil) and the N-methyl-D-aspartate (NMDA) antagonist memantine, offer only symptomatic relief. While these drugs improve memory and daily functioning, they fail to provide a cure or halt the disease’s progression [23]. The imperative to identify novel targets and tools for AD prevention and modification arises from the elusive nature of the disease’s etiology. While only a tiny fraction of AD cases, approximately 5%, are attributed to genetic mutations, the remaining 95% constitute sporadic cases of unknown origin [23]. This enigma has led to a shifting research focus towards prevention rather than treatment, driven by the staggering socioeconomic burden imposed by AD. Projections indicate a substantial rise in diagnosed cases, making it crucial to explore innovative strategies. Delaying AD onset even by just one year can significantly reduce the incidence of new cases and alleviate the escalating financial burden [24]. In this context, the Mediterranean diet emerges as a promising avenue, supported by epidemiological and clinical studies highlighting its remarkable impact on cognitive health [25]. Higher adherence to the Mediterranean diet not only reduces the risk of developing mild cognitive impairment (MCI) and AD, but also demonstrates the potential to mitigate the progression from MCI to AD, positioning it as a powerful preventive strategy against neurodegenerative disorders, including AD [26]. Moreover, recent guidelines from the World Health Organization (WHO) have endorsed the Mediterranean diet to decrease the risk of cognitive decline and dementia [27].

**Figure 1:**
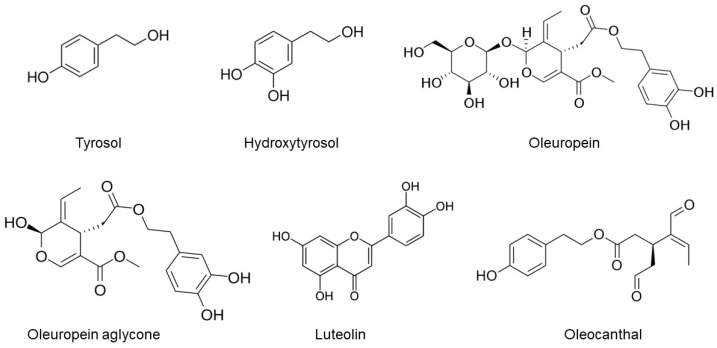
The chemical structures of selected phenolic compounds in EVOO.

Central to the Mediterranean diet’s neuroprotective arsenal is extra-virgin olive oil (EVOO), a cornerstone of this dietary regimen that is characteristically abundant in Mediterranean populations [28]. EVOO, which constitutes a substantial portion of the daily fat intake, stands out as a source of over 100 distinct phenolic compounds, including secoiridoids such as oleuropein aglycone (OLG), oleuropein (OLE), oleocanthal (OC), oleacein (known as hydroxy OC; OH-OC), tyrosol, and hydroxytyrosol (HT), as shown in Figure 1 [29]. EVOO, derived from the initial pressing of olive fruit by mechanical means, comprises approximately 95% glycerol and 5% nonglycerol components [30]. These phenolic compounds’ anti-inflammatory, antioxidant, and neuroprotective properties have garnered significant attention for their potential role in preserving cognitive function and combatting AD [29,31,32].

This review aims to unravel the beneficial effects of EVOO and its phenolic compounds against AD. Regarding the first aim, the promising avenues for prevention and disease modification offered by EVOO and its bioactive constituents are elucidated based on preclinical and clinical findings. Then, the molecular mechanisms underlying the neuroprotective properties of EVOO and its bioactive constituents are presented to comprehensively understand how EVOO intervention may influence AD pathogenesis.

In preparing this review, we systematically searched the PubMed and Google Scholar databases for articles published from 1999 to 2023. This search strategy incorporated key terms, such as EVOO, phenolic compounds, and Alzheimer’s disease, to capture studies that specifically addressed the biochemical or therapeutic effects of EVOO or its constituents on AD. The inclusion criteria targeted research articles that provided insights into the impact of EVOO on AD’s pathological markers or progression, whether through in vitro, in vivo, or clinical investigations. Articles focusing on non-neurological aspects of EVOO were excluded from the review to maintain a clear focus on AD. Our aim was to present a balanced overview to ensure a broad representation of the current research landscape.

## 2. EVOO and Its Phenolic Compounds

EVOO is renowned for its culinary virtues and its rich array of phenolic compounds, which are garnering increasing attention for their potential therapeutic implications in AD [30,33,34]. Central to this group of compounds are primary phenolics like OLE, HT, and tyrosol, which undergo dynamic changes during olive maturation and oil extraction, influencing EVOO’s health properties [30,35,36]. OLE, a major phenolic in olive fruit and its derivative OLG, and HT, known for its potent antioxidant capacity, possess critical neuroprotective effects [34,36,37]. The minor polar compounds in EVOO, including the secoiridoids OC and OH-OC, and phenolic alcohols such as tyrosol and HT, add to this protective arsenal. These compounds, notable for their antioxidant and anti-inflammatory properties, are essential in addressing the oxidative stress and chronic neuroinflammation characteristic of AD [33,36,37]. Additionally, variants like deacetoxy OLE and ligstroside aglycone (Figure 1), along with other constituents like phenolic acids, flavonoids, and lignans (exemplified by pinoresinol and acetoxypinoresinol), contribute to EVOO’s overall antioxidant capacity [34,37]. This intricate network of phenolic compounds plays a pivotal role in neutralizing reactive oxygen species (ROS) and protecting neuronal cells from oxidative damage, thereby impacting the progression of AD [35,36]. The diverse spectrum of EVOO’s phenolic content underscores its potential as a natural therapeutic agent in the intricate pathology of AD, highlighting the need for continued research into its nuanced role in neuroprotection and disease mitigation [30,33,34,35,36,37].

## 3. EVOO and Phenolic Compounds: Modulating the Hallmarks of AD

Central to AD pathology are Aβ plaques resulting from the abnormal accumulation and aggregation of Aβ peptides in the brain [38]. These plaques, predominantly located in memory-critical areas such as the cerebral cortex and hippocampus, disrupt neuronal communication, induce oxidative stress, and trigger inflammatory responses [5,38,39]. Complementing the Aβ plaque pathology is the formation of tau protein tangles [40]. Tau, a microtubule-stabilizing protein, undergoes hyperphosphorylation, detaining from microtubules and forming intracellular NFTs [40,41]. This tau pathology contributes to impaired neuronal transport, cell dysfunction, and death, closely correlating with the cognitive decline observed in AD patients [42,43].

**Figure 2:**
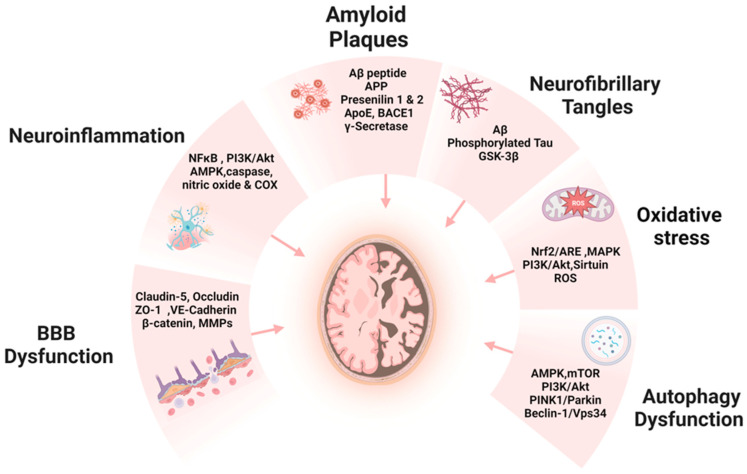
Fundamental pathological features and molecular mechanisms implicated in the progression of AD, revealing critical targets for therapeutic ligand intervention. Abbreviations: nuclear factor kappa-light-chain-enhancer of activated B cells (NFκB), phosphoinositide 3-kinases/protein kinase B (PI3K/Akt), AMP-activated protein kinase (AMPK), cyclooxygenase (COX), zonula occludens-1 (ZO-1), vascular endothelial cadherin (VE-Cadherin), matrix metalloproteinases (MMPs), amyloid beta (Aβ), amyloid precursor protein (APP), apolipoprotein E (APOE), beta-site APP cleaving enzyme 1 (BACE1), glycogen synthase kinase-3 beta (GSK-3β), nuclear factor erythroid 2-related factor 2/antioxidant response element (Nrf2/ARE), mitogen-activated protein kinases (MAPK), reactive oxygen species (ROS), AMP-activated protein kinase (AMPK), mammalian target of rapamycin (mTOR), phosphoinositide 3-kinases/protein kinase B (PI3K/Akt), PTEN-induced kinase 1/Parkin (PINK1/Parkin), vacuolar protein sorting 34 (Vps34).

Further complicating this scenario is the dysfunction of the BBB. In AD, the BBB’s selective permeability is compromised, allowing the infiltration of neurotoxic substances and inflammatory agents into the brain, thereby exacerbating neuronal damage [10,44]. AD is also marked by chronic neuroinflammation, primarily driven by the immune system’s response to Aβ plaques and tau tangles [39,45]. Activated microglia release pro-inflammatory cytokines, perpetuating inflammation and neuronal damage [45]. Neuroprotective mechanisms in AD, which involve preserving neuronal structure and function, preventing neuron loss, and enhancing neurogenesis and synaptic plasticity, are essential yet often compromised [46]. Oxidative stress, characterized by an imbalance between free radicals and antioxidants, leads to extensive neuronal damage through mechanisms like lipid peroxidation and DNA damage [47,48]. Additionally, the regulation of autophagy, a critical process for recycling damaged cellular components, is disrupted in AD. Dysregulated autophagy contributes to the accumulation of toxic proteins such as Aβ and tau, exacerbating neurodegenerative processes, as shown in Figure 2 [49,50].

In this complex landscape, EVOO and its phenolic compounds emerge as multifaceted players with potential influences on these key pathological hallmarks [34,51]. These compounds modulate the formation and accumulation of Aβ plaques and tau protein tangles [31,52]. They aid in disrupting the aggregation of Aβ peptides, potentially enhancing their clearance from the brain, and mitigating associated disruptions in neuronal communication and oxidative stress [53,54,55,56,57]. Simultaneously, they interfere with the hyperphosphorylation of tau proteins, thereby preventing the formation of NFTs and preserving crucial neuronal transport systems [52,58]. EVOO’s role extends to maintaining the integrity of the BBB, potentially fortifying its selective permeability, and limiting the infiltration of neurotoxic substances [59,60]. EVOO’s phenolic compounds offer anti-inflammatory benefits, possibly reducing chronic neuroinflammation by downregulating pro-inflammatory cytokines [57,59,61]. Moreover, EVOO’s rich array of antioxidants directly counteract the oxidative stress prevalent in AD. In regulating autophagy, EVOO contributes to the clearance of toxic proteins like Aβ and tau, thereby mitigating some neurodegenerative processes [62]. Therefore, EVOO and its phenolic compounds collectively suggest their potential as part of a dietary strategy for managing or preventing AD.

## 4. In Vitro and In Vivo Preclinical Studies

In the following section, we will delve into in vitro and in vivo preclinical studies focusing on the use of EVOO and its phenolic compounds in cell culture and AD mouse and rat models. These studies are pivotal in understanding how EVOO might combat AD-related pathologies.

### 4.1. In Vitro Studies

Several in vitro studies have provided insightful revelations about the neuroprotective and antioxidant properties of EVOO phenolic extracts and various individual phenolic compounds found in EVOO and their potential benefits against neurodegenerative diseases like AD. A recent study by Barbalace and colleagues (2021) has demonstrated the protective effects of EVOO extracts on differentiated SH-SY5Y neuroblastoma cells subjected to peroxide-induced oxidative stress [63]. The cells were pretreated with 10 µg/mL of EVOO extracts 24 h prior to the oxidative challenge. This protection is notably attributed to the upregulation of key antioxidant enzymes, including heme oxygenase 1 (HO-1), glutathione reductase, thioredoxin reductase 1, and NADPH quinone oxidoreductase 1. These findings not only highlight the significant antioxidant properties of EVOO extracts but also shed light on their role in regulating proteins associated with neuronal plasticity. The ability of EVOO to mitigate oxidative stress and bolster neuronal health indicates its potential as a therapeutic agent in neuroprotection. The upregulation of antioxidant enzymes points to a targeted mechanism through which EVOO exerts its protective effects, reaffirming the importance of its phenolic compounds in maintaining neuronal integrity and function [63].

In another study, Hornedo-Ortega et al. (2018) explored the effects of HT and resveratrol on neuroinflammation mediated by microglia, the brain’s resident immune cells [64]. Both compounds inhibited the nuclear translocation of Nuclear factor kappa B (NFκB), a critical factor in inflammation, thereby reducing the production of pro-inflammatory agents like interleukin-6 (IL-1β), inducible nitric oxide synthase (iNOS), cyclooxygenase-2 (COX-2), and tumor necrosis factor-alpha (TNF-α). The action of these compounds contributes to the shift of microglia from an inflammatory state to a neuroprotective one, suggesting the potential of these compounds as adjunct therapies in treating neurodegenerative diseases [64]. Notably, in a study by Maiuri et al. (2005), HT was used at a high concentration (200 μM) to inhibit iNOS and COX-2 expression in lipopolysaccharide (LPS)-stimulated J774 cells by preventing the activation of NFκB, STAT-1α, and IRF-1 [65]. Moreover, Richard et al. (2011) reported that HT inhibited the production of NO and PGE2 with an IC50 of 11.4 and 19.5 μM, respectively, in LPS-stimulated RAW 264.7 cells [66]. Additionally, these authors observed reduced cytokine and chemokine secretion via the NFκB pathway. Takeda et al. (2014) corroborated these findings with similar results, and Bigagli et al. (2017) further demonstrated that nutritionally relevant concentrations of HT and OLE (50 and 10 μM) in LPS-treated RAW 264.7 cells resulted in HT at 10 μM inhibiting the production of NO and PGE2 and inducing Nrf2 nuclear translocation [67,68]. Furthermore, HT modulated cytokine signaling, particularly inhibiting the SOCS and JAK-STAT pathways, and promoted the production of the anti-inflammatory cytokine interleukin-10 (IL-10). OLE has also been noted for its ability to attenuate microglial activation, primarily through modulating NFκB activation [66]. These findings support the potential of HT and OLE as effective treatments for neurodegenerative conditions [64].

Another study confirmed the antioxidant and cytoprotective effects of HT and its metabolites homovanillyl alcohol-4′-O-glucuronide and homovanillic acid-4′-O-glucuronide at dietarily achievable concentrations in neuroblastoma SH-SY5Y and neuronal-like LUHMES cells, supporting the practical implications of dietary EVOO consumption for neuronal health [69]. Another in vitro study investigated the effects of OLG and HT on autophagy in SH-SY5Y cells [62]. The combination of OLG and HT, evaluated at different molar ratios with an overall concentration of 75 μM, synergistically activated autophagic flux, aiding in preventing cell damage caused by Aβ42 oligomers, reducing ROS production, and mitigating mitochondrial impairment [62].

Researchers also examined the impact of various olive polyphenols on mitochondrial function in SH-SY5Y cells expressing APP695 (SH-SY5Y-APP), a cellular model of AD [70]. The study tested different purified phenolic secoiridoids at a concentration of 0.05 µM. The phenolic compounds significantly increased basal ATP levels, with some compounds, such as OC and ligstroside, enhancing the capacity of respiratory chain complexes and influencing the expression of genes related to mitochondrial biogenesis and antioxidative capacity [70].

Findings from additional in vitro studies on OC have highlighted the significant role of OC in combating hallmarks of AD and related tauopathies [71,72]. Treatment with OC induced changes in the secondary structure of the tau-441 protein and disrupted its aggregation process [73], which suggests the potential of OC to reduce NFT formation. To clarify the mechanism, further investigation into the interaction between OC and the tau protein’s fibrillogenic fragment K18 under biomimetic conditions showed that OC can covalently modify K18 by forming a Schiff base with lysine residues. This modification inhibits the fibrillization of tau [73]. These findings were further confirmed by Li and colleagues (2009), who extended these findings by demonstrating that OC maintains tau protein in a naturally unfolded state, thereby inhibiting its fibrillization [72]. This inhibition was achieved by forming an adduct between OC and the lysine residue in the tau sequence through the two aldehyde groups in OC without disrupting the normal function of the tau protein [72]. Together, these studies illuminate the multifaceted roles of OC in neuroprotection.

Other studies have also demonstrated the neuroprotective effects of OC [74,75]. In this work, the investigators unraveled the mechanisms by which OC influences AD risk, emphasizing its capability to enhance the clearance of Aβ from the brain. Central to both investigations was the examination of OC’s effect on crucial Aβ transport proteins across the BBB, namely, P-glycoprotein (P-gp) and low-density lipoprotein receptor-related protein 1 (LRP1). Utilizing mouse brain endothelial bEnd3 cells, OC treatment for 72 h significantly increased the expression and function of P-gp and LRP1 in a concentration-dependent manner in the 0.5–50 µM range. Additionally, the findings provided insights into the impact of OC on Aβ degradation, where OC significantly enhanced the degradation of Aβ_40_, which can be attributed to the increased activity of Aβ-degrading enzymes, namely, insulin-degrading enzyme (IDE) and neprilysin. These results suggest a dual action of OC by promoting the clearance of Aβ from the brain across the BBB and stimulating its degradation, thus supporting the protective effect of OC consumption against AD [74].

In addition, the effect of OC on attenuating the toxic effect of Aβ oligomers (Aβos) in the CCF-STTG1 human astrocytoma cell line was evaluated [31]. In these experiments, CCF-STTG1 cells were treated with 100 nM Aβo, 5 μM OC, or a combination of 100 nM Aβo and 5 μM OC for 3 or 7 days. The results demonstrated that OC effectively shielded neurons from Aβo-induced synaptic protein reduction, mainly SNAP-25 and PSD-95, and mitigated Aβo-triggered inflammation by astrocytes. This effect was evidenced by a decrease in IL-6 and the upregulation of GFAP due to OC treatment. Moreover, OC preserved the neuro-supportive functions of astrocytes by preventing the downregulation of critical transporters like GLT1 and GLUT1. These findings reinforce the protective effect of OC against AD pathology, operating through mechanisms that reduce inflammation and maintain cellular functions in the brain. The results add to the growing body of evidence supporting the beneficial impact of OC on brain health and open new avenues to explore OC’s direct effects on neuronal cells, independent of astrocyte-mediated protective interactions [31].

The effects of OLG and HT on Aβ aggregation have also been investigated [76]. The research utilized biophysical methods and cell biology techniques to understand how these compounds influence the fibrillation of Aβ_42_ (25 µM). The findings revealed that OLG prevents the formation and growth of toxic Aβ_42_ oligomers into mature fibrils by interacting with the peptide’s N-terminus, while HT accelerates the formation of non-toxic fibrils. Both polyphenols, at a concentration of 75 µM each, were shown to stabilize Aβ42 oligomers and fibrils, reducing their seeding activity (with the seed concentration at 1.25 µM relative to the monomer concentration) and neurotoxic effects in human neuroblastoma SH-SY5Y cells. These results highlight the potential of OLG and HT in AD prevention and therapy, suggesting their ability to mitigate amyloid-related neurodegeneration [76].

An in vitro study investigated the neuroprotective and anti-inflammatory effects of a triterpenoid-enriched fraction from olive leaves [77]. The fraction was obtained through supercritical fluid extraction (SFE) and dynamic adsorption/desorption using sea sand as an adsorbent. The study examined the effect of this fraction, tested at two different concentrations (20 and 40 μg/mL) for 24 h, on the SH-SY5Y cell line. A comprehensive lipidomics analysis was conducted using charged-surface hybrid chromatography-quadrupole-time-of-flight mass spectrometry (CSH-Q-TOF MS/MS) [77]. This advanced analytical approach, coupled with freely available lipidomic annotation tools and databases, facilitated the annotation of over 250 intracellular lipids. Using bioinformatics and statistical tools, the study identified significant increases in specific phospholipids, particularly phosphatidylcholines and phosphatidylethanolamines, which protected the cells against death caused by Aβ42. Furthermore, the study noted a decrease in several triacylglycerols within the cells, indicating that triterpenoids from olive leaves may act as promising neuroprotective agents [77]. Indeed, additional studies are required to further validate and clarify the neuroprotective capabilities and mechanisms of olive leaf-derived triterpenoids.

Besides its protective effects in AD, in Parkinson’s disease research, hydroxytyrosol butyrate (HT-B), a polyphenol derivative from olives, has been shown to offer significant neuroprotection [78]. Specifically, HT-B effectively inhibited apoptosis in SH-SY5Y neuronal cells exposed to 6-hydroxydopamine (6-OHDA), a toxin closely related to Parkinson’s disease pathology. In this study, SH-SY5Y cells were co-treated with HT-B at concentrations of 5 and 10 μM for 6 h with 6-OHDA (100 μM), having been pretreated with 6-OHDA for 12 h. The observed neuroprotective action involved the induction of the transcription factor nuclear factor erythroid 2-related factor 2 (Nrf2). Upon activation by HT-B, Nrf2 initiates a cascade of protective responses via upregulating the expression of HO-1, an enzyme pivotal in mitigating oxidative stress. This upregulation of HO-1 is crucial for reducing the oxidative damage and apoptosis typically triggered by 6-OHDA. Furthermore, HT-B’s effectiveness is enhanced by its fat solubility, which facilitates its interaction with critical cellular components. The study also identified specific cysteine residues in Kelch-like ECH-associated protein 1 (Keap1) that are vital for Nrf2 activation by HT-B, underlining the compound’s targeted approach in combating neurotoxicity [78].

Another study by Visioli et al. (2022) confirmed the neuroprotective effects of HT and HT-B in in the cell line 7PA2, a cell model for Aβ toxicity with mitochondrial dysfunction observed in AD [79]. While both compounds were neuroprotective, unlike the HT-B effect mediated by NRF2 activation, HT (5 μM, 8 h) was found to be effective in correcting the mitochondrial energetic dysfunction in 7PA2 cells by boosting ATP levels and enhancing the number and fusion of mitochondria. These findings collectively emphasize the varied therapeutic possibilities of HT and HT-B in treating neurodegenerative diseases by addressing both oxidative stress and mitochondrial dysfunction [79].

Recent studies have also reported the anti-inflammatory effects of olive oil extracts in neurodegenerative disease models [80]. Specifically, EVOO phenolic extracts prepared from the Moraiolo cultivar (MVOO-PE) and EVOO demonstrated potent activity against neuroinflammation in LPS-stimulated BV2 microglia cells in a concentration-dependent manner ranging between 1 and 20 µg/mL. In addition, both MVOO-PE and EVOO extract effectively reduced cell death and attenuated the activation of the toll-like receptor 4 (TLR4)/NOD-like receptor pyrin domain-containing-3 (NLRP3) signaling pathway, leading to a decrease in vital inflammatory markers, such as TLR4, NFkB, the NLRP3 inflammasome, IL-1β, the COX-2 isoenzyme, and ionized calcium-binding adaptor molecule 1 (Iba-1). Furthermore, MVOO-PE uniquely demonstrated an ability to downregulate mRNA expression of various inflammatory mediators and suppress cytokine secretion [80]. Taken together, MVOO-PE and EVOO extract could reduce neuroinflammation by targeting the TLR4/NLRP3 axis pathway.

### 4.2. In Vivo Preclinical Studies

Several studies, including ours, have evaluated the neuroprotective effects of EVOO and its phenolics in AD mouse models. Our group’s research focused on exploring the impact of an EVOO-enriched diet on AD and cerebral amyloid angiopathy (CAA) in TgSwDI mice [52]. In this study, mice were fed a 0.7 g/day dose of an EVOO-enriched diet for 3 months (starting the treatment at 4 months of age—treatment mode) and 6 months (starting the treatment at 1 month of age—preventive mode). The findings demonstrated that feeding mice with EVOO for 6 months, beginning before the onset of Aβ accumulation, significantly reduced total Aβ and tau levels in the brain. A marked improvement in cognitive behavior accompanied this effect. This effect was mediated by the upregulation of Aβ clearance pathways and the downregulation of Aβ production pathways. Furthermore, the study revealed that the shorter duration of EVOO consumption, for 3 months, starting after Aβ accumulation, was also associated with improved Aβ clearance and reduced Aβ levels, an effect that was partially attributed to the increased expression of P-gp and LRP1. Additionally, EVOO consumption increased Aβ clearance by inducing the APOE-dependent pathway, as demonstrated by increased expressions of ATP-binding cassette transporter A1 (ABCA1) and APOE. This alteration in the APOE pathway was mediated by the activation of nuclear receptors, specifically the peroxisome proliferator-activated receptor gamma (PPARγ) and liver-X receptors (LXRs) [52].

Lauretti et al. (2017) investigated the effects of an EVOO-enriched diet in a triple transgenic (3xTg) mouse model expressing both Aβ and tau pathologies [81]. 3xTg mice (6 months old) were fed either a standard or an EVOO-enriched diet for 6 months. The mice underwent behavioral tests at 6 months (for baseline assessment), 9 months (after 3 months of treatment), and 12 months (after 6 months of treatment) of age. The findings of this study demonstrated notable improvements in the mice that consumed the EVOO-enriched diet. These improvements included enhanced behavioral performance, increased synaptic integrity, and reduced insoluble Aβ peptides and Aβ deposition. Additionally, there was a noticeable decrease in tau phosphorylation. These positive outcomes are attributed to several mechanisms triggered by EVOO. Firstly, the reduction in Aβ brain load was linked to a shift in APP processing from the amyloidogenic towards the nonamyloidogenic pathway. Reduced Aβ was evidenced by the increase in β-secretase (BACE1), soluble APP-β (sAPP-β), and α-secretase (ADAM10) levels in the EVOO-treated mice. Furthermore, the addition of EVOO stimulated autophagy in brain cells, supported by higher reactivity of the autophagy markers autophagy related 5 (ATG5) and ATG7 in the brain cortexes of the EVOO-treated mice compared to the control group. Autophagy activation is crucial in reducing harmful protein accumulations and maintaining neuronal health. In addition, the EVOO-treated mice exhibited less phosphorylation of tau protein at specific sites (Ser202/Thr205 and Ser396/Ser404) and reduced microglia activation. These findings collectively highlight the neuroprotective effects of EVOO [81].

Other studies, such as the one conducted by Grossi et al. (2013), have reported the neuroprotective effects of individual olive-derived phenols in AD mouse models [82]. In their study, the researchers examined the effects of dietary supplementation with OLG (50 mg/kg of diet) for 8 weeks in young (1.5-month-old) and aged (4-year-old) TgCRND8 mice, a model for AD. The investigators found that OLG treatment notably counteracted the neurotoxic effects of Aβ and Aβ-induced cognitive impairment. This effect was evidenced by a reduction in Aβ plaque load due to enhanced autophagy, which restored the lysosomal system and activated microglia to migrate to Aβ deposits for plaque disassembly. These findings elucidate the protective effects of OLG against age-related and AD-type neurodegeneration. The results of Grossi et al.’s study reinforce the potential therapeutic role of OLG in preventing or delaying the onset of AD and reducing its severity. The study highlights the importance of dietary supplementation with olive-derived phenols like OLG as a potential strategy for combating AD [82].

Recently, we reported that oleuropein-rich olive leaf extract added to the diet of 5xFAD mice at a dose of 695 μg/kg body weight/day for 3 months, starting at 3 months of age, effectively reduced Aβ burden in mouse brains, as demonstrated by reduced brain total Aβ deposits and soluble Aβ levels promoted by the increased expression of Aβ clearance proteins P-gp and LRP1 and the induced nonamyloidogenic pathway [83]. In addition, the oleuropein-rich olive leaf extract-enriched diet significantly reduced neuroinflammation by suppressing astrocyte and microglia activation, as evidenced by reduced GFAP and Iba1 levels, and attenuated the production of pro-inflammatory cytokines IL-1β and IL-6 by inhibiting the NF-κB and NLRP3 inflammasome pathways. This extract also reduced the expressions of Receptor for Advanced Glycation End-products (RAGE) and High Mobility Group Box 1 (HMGB1), contributing to decreased inflammation. As an outcome of reduced Aβ and neuroinflammation, the oleuropein-rich olive leaf extract-enriched diet enhanced synaptic markers and improved memory performance in 5xFAD mice, indicating its potential to prevent or slow AD progression [83].

Another line of research investigated OC-rich EVOO in the AD mouse model TgSwDI [59]. TgSwDI mice at the age of 6 months received a daily EVOO-OC dose of 476 μg/kg body weight for 3 months. The OC-rich EVOO consumption restored BBB function and reduced AD-related pathology. These effects were primarily mediated by two fundamental mechanisms. First, consistent with the above-described findings, OC-rich EVOO demonstrated a capability to reduce neuroinflammation by inhibiting the activation of NLRP3 inflammasomes, a critical component in the inflammatory response associated with AD. Second, the study revealed that OC-rich EVOO reduced brain Aβ load by promoting autophagy via the activation of the AMP-activated protein kinase (AMPK)/Unc-51-like autophagy activating kinase 1 (ULK1) pathway. These findings, collectively, suggested the dietary supplementation with OC-rich EVOO as a beneficial strategy to slow down or potentially halt the progression of AD, primarily through its dual action of restoring BBB function, reducing neuroinflammation via suppressing the NLRP3 inflammasome pathway, and promoting autophagy via the AMPK/ULK1 pathway [59].

In another comprehensive study on the protective effect of OC, wild-type and 5xFAD mouse models were utilized to investigate the complex relationship between aging (4 and 9 months old), AD, and metabolic-behavioral changes without and with OC administered at 10 mg/kg for 3 months [84]. The Promethion cage system^®^ was used to monitor changes in physiological, metabolic, and behavioral parameters with age and AD pathology. The findings revealed significant alterations in body weight, food and water intake, energy expenditure, dehydration, and respiratory exchange rate with age and AD pathology. Furthermore, marked disruptions in sleep patterns and an increase in anxiety-like behavior were observed. These metabolic and behavioral changes were more severe in the presence of AD pathology compared to normal aging, suggesting a direct link to Aβ deposition and BBB disruption. The findings also indicated a novel inverse correlation between sleep duration and BBB breakdown, highlighting the intricate relationship between sleep disturbances and AD progression. The treatment of 5xFAD mice with OC significantly improved the altered metabolic and behavioral parameters. Notably, OC effectively restored metabolic parameters and alleviated anxiety-like behavior and sleep disturbances. These outcomes suggest that OC counteracts the metabolic and behavioral disruptions associated with AD [84]. The beneficial effects of OC and ligstroside have also been confirmed in an aging mouse model, namely, NMRI mice [70]. In this study, NMRI mice aged 12 months received a diet supplemented with OC or ligstroside at a dose of 6.25 mg/kg body weight for 6 months. Supplementing the mice’s diet with OC or ligstroside improved spatial working memory, restored brain ATP levels, and significantly extended lifespan. These findings highlight the positive impact of OC and ligstroside on mitochondrial bioenergetics and cognitive function in aging [70].

A comparison study of 5xFAD mice fed with OC or EVOO low in OC (OC-low EVOO) was performed to clarify and compare the specific effect of OC against AD [57]. In this study, 3-month-old 5xFAD mice were fed equivalent doses of OC-low EVOO (0.5 mg total phenolic content/kg) and OC (0.5 mg OC/kg) for 3 months. The findings demonstrated that OC and OC-low EVOO effectively reduced brain Aβ levels, astrocyte activation, and neuroinflammation. This effect was linked to the suppression of NLRP3 inflammasome activation and NFκB pathways. However, OC uniquely suppressed the RAGE/HMGB1 pathway, indicating a potentially more substantial anti-neuroinflammatory effect than OC-low EVOO. Both OC-low EVOO and OC improved the integrity of the BBB by upregulating the tight and adherence junction proteins claudin5, occludin, and ve-cadherin, contributing to enhanced BBB function. In terms of Aβ pathology, both treatments reduced brain total Aβ levels, Aβ deposits, and soluble Aβ40 and Aβ42. To explain the reduced brain Aβ, both OC-low EVOO and OC increased the expression of Aβ transport proteins P-gp and LRP1, indicating improved clearance of Aβ across the BBB, and both shifted APP processing toward the nonamyloidogenic pathway. These findings suggest that OC-low EVOO and OC are both effective and support that diet supplementation with EVOO or OC could protect against AD [57].

A study by Rigacci and colleagues (2015) reported the molecular and cellular mechanisms by which OLE induces autophagy in neurodegeneration using in vitro and in vivo models [85]. The authors found that OLE triggered autophagy in vitro in neuroblastoma cells by activating the calcium (Ca^2+^)-calmodulin-dependent protein kinase kinase β (CAMKKβ)–AMPK signaling pathway. Specifically, OLE induced a rapid release of Ca^2+^ from the sarcoplasmic reticulum stores in cells. This release of Ca^2+^ activated CAMKKβ and the phosphorylation and activation of AMPK. These findings were further confirmed in vivo in 4- and 10-month-old TgCRND8 mice fed a diet supplemented with OLE (50 mg/kg of diet/day) for 8 weeks. The findings also demonstrated a link between AMPK activation and the inhibition of mechanistic Target of Rapamycin (mTOR), a critical regulator of cell growth and metabolism that negatively regulates autophagy. The researchers observed decreased phosphorylation of mTOR and its substrate, p70-S6 kinase, along with increased phosphorylation of AMPK. This pattern suggested that the autophagy activation by OLE is mediated by the inhibition of mTOR [85].

In vivo studies have also demonstrated the neuroprotective and anti-inflammatory benefits of HT added to the diet of AD mouse models [86,87]. TgCRND8 mice fed a low-fat diet supplemented with HT for 8 weeks, starting at 4 months, exhibited reduced brain Aβ_42_ and pE3-Aβ plaques. These changes in the HT study were accompanied by a marked reduction in TNF-α expression and astrocyte reactivity, indicating an anti-inflammatory effect. This effect was associated with improved memory in TgCRND8 mice. The study also uncovered that the beneficial effects of HT could be explained by the induction of macroautophagy and the modulation of MAPK signaling pathways, both involved in Aβ clearance and reduced inflammation and cell death. These results confirm the neuroprotective effects of HT, as evidenced by improved memory, reduced Aβ plaque burden, and decreased inflammation via the modulation of macroautophagy and MAPK signaling pathways [87].

Furthermore, in an APP/PS1 mouse model of AD, HT treatment at 5 mg/kg/day for 6 months modulated mitochondrial oxidative dysfunction, a critical factor in AD progression [86]. HT improved mitochondrial health by reducing mitochondrial carbonyl proteins and oxidized glutathione while increasing superoxide dismutase expression and restoring phase II enzymes involved in detoxification and antioxidant defenses. The study also noted reduced brain pro-inflammatory factors through modulating the MAPK signaling pathway [86]. These results underscore the significant neuroprotective effects of HT by enhancing mitochondrial health and reducing oxidative stress and inflammation.

To investigate EVOO as a medical food, the synergistic effects of combining OC-rich EVOO with donepezil, an AD medication, were evaluated in 5xFAD mice [31]. As expected, the noncholinergic mechanisms of donepezil, administered at 1 mg/kg/day for 1 month, were significantly enhanced in 4-month-old 5xFAD mice fed with EVOO that reduced brain Aβ load. This reduction was achieved via enhancing Aβ clearance pathways across the BBB, enzymatic degradation, and shifting APP processing towards the nonamyloidogenic pathway. Additionally, there was an upregulation of synaptic proteins and a decrease in neuroinflammation [31]. The in vitro and in vivo studies are summarized in Table 1.

## 5. Clinical Studies

The protective effects of EVOO in humans were evaluated either as part of the Mediterranean diet or when ingested as olive oil, as summarized in Table 2. The Italian Longitudinal Study on Aging (ILSA), conducted by Solfrizzi et al. (1999), assessed the effect of diet on changes in cognitive functions using standardized tests and a semi-quantitative food frequency questionnaire. This study was part of a larger multicenter, population-based cohort study designed to evaluate age-related diseases and physiologic and functional changes in the aging process. The ILSA study included both survey and prospective components and involved a sample of 5632 subjects, aged between 65 and 84 years, who were free-living or institutionalized. The subjects were randomly selected from the electoral rolls of eight municipalities in Italy, stratified by age and sex. The data for this study were obtained from a prevalence survey carried out in Casamassima (Bari, Southern Italy) between March 1992 and June 1993. The final study population consisted of 278 free-living elderly subjects who underwent both the neuropsychological evaluation and dietary assessment. The findings from the ILSA study revealed an inverse relationship between the intake of monounsaturated fatty acids (MUFAs) and cognitive decline in individuals with low MMSE scores, suggesting a protective effect of high MUFA intake against cognitive decline [88].

Moreover, in a systematic review aimed at determining the association between the Mediterranean diet and cognitive impairment, specifically MCI and AD [89], a comprehensive search through major databases indicated that higher adherence to the Mediterranean diet significantly reduced the risk of developing MCI and AD. Individuals with the highest level of adherence had a 33% lower risk of cognitive impairment compared to those with the lowest level of adherence [89]. These results suggest that consuming olive oil as part of the Mediterranean diet could reduce the risk of dementia or slow the conversion of MCI to AD.

Tzekaki and colleagues (2021) investigated the potential therapeutic effects of EVOO in MCI patients [90]. This study, part of the MICOIL clinical trial, included participants from the Mediterranean area, namely, the Thessaloniki region (northern Greece). All subjects underwent comprehensive neuropsychological assessments, including the Mini-Mental State Examination (MMSE) and the Alzheimer’s Disease Assessment Scale—Cognitive sub-scale (ADAS-cog) [90]. The participant group comprised 80 individuals aged 64–90, divided into the following sub-groups: MCI patients consuming 50 mL EVOO daily, MCI patients not consuming EVOO, AD patients, and healthy individuals used as a reference. The MCI patients were further divided based on the duration of EVOO consumption (1 month and 12 months) [90]. The authors revealed that EVOO consumption over 12 months led to increased levels of the neuroprotective protein BMI1 concurrent with reduced levels of the tumor suppressor gene p53 in serum. These changes in biomarkers indicate a reversal of some AD pathological markers in response to EVOO consumption.

Furthermore, the authors observed that EVOO treatment modulated blood AD-related biomarkers, including phosphorylated tau (p-tau), Aβ42, and the Aβ42/Aβ40 ratio. Additionally, this study demonstrated that the upregulation of BMI1 induced by EVOO is associated with reduced oxidative stress and inflammatory responses, critical contributors to AD pathology. The correlation between BMI1 upregulation and a decrease in these detrimental processes suggests a protective mechanism triggered by EVOO consumption [90].

In the MICOIL pilot study, Tsolaki et al. (2020) conducted a randomized clinical trial to compare the effects of Greek high-phenolic early-harvest extra-virgin olive oil (HP-EH-EVOO), moderate-phenolic EVOO (MP-EVOO), and the Mediterranean diet (as the control) in individuals with MCI. The study involved a comprehensive neuropsychological battery administered at baseline and after 12 months to assess cognitive performance. The results showed that Group 1 (HP-EH-EVOO) exhibited better cognitive performance compared to both Group 2 (MP-EVOO) and Group 3 (Mediterranean diet) across almost all cognitive domains—specifically, Groups 1 and 2 showed significantly improved ADAS-cog and MMSE scores. Group 2 (MP-EVOO) also showed a significant improvement compared to Group 3 (Mediterranean diet) in these cognitive tests. Furthermore, the study revealed that the cognitive improvements associated with HP-EH-EVOO and MP-EVOO occurred regardless of the participants’ genetic predisposition to AD with regard to the presence of the APOEɛ4 allele. Collectively, the findings from the MICOIL pilot study indicate that the long-term intervention with HP-EH-EVOO or MP-EVOO could improve cognitive function compared to adherence to the Mediterranean diet alone, as evidenced by the ADAS-cog and MMSE tests, and the effect could be independent of the presence of the APOEɛ4 allele [91]. Dimitriadis et al. (2021) extended this research by investigating the neurological impacts of these interventions using electroencephalography (EEG) resting-state recordings [92]. The authors found that HP-EH-EVOO consumption caused changes in brain connectivity patterns, including reduced signal spectrum within the 1–13 Hz range and a decreased theta/beta ratio during eyes-open conditions. These results indicated enhanced brain connectivity and reduced over-excitation of information flow, suggesting that HP-EH-EVOO might mitigate cognitive impairment in MCI individuals by improving brain network functionality [92].

In a recent double-blinded pilot study involving 25 female and male participants with MCI, aged between 55 and 75 years, the daily consumption of 30 mL of EVOO for six months enhanced brain functional connectivity, reduced BBB permeability, and improved memory [60]. EVOO’s effect on the BBB was evidenced by its increased restrictive ability to prevent Gadolinium (Gd), a contrast molecule with limited brain access, from permeating it. In contrast, while those who consumed refined olive oil (ROO) showed improved memory and increased brain activation in fMRI tasks, ROO did not alter BBB function. Both the EVOO and ROO groups demonstrated significant memory improvements, notably in Clinical Dementia Rating (CDR) scores. Both olive oil types significantly influenced AD blood biomarkers by reducing the Aβ_42_/Aβ_40_ and p-tau/t-tau ratios. These results suggest that, aside from the health benefits attributed to EVOO’s phenolic compounds, monounsaturated fats, such as oleic acid, major components in EVOO and ROO, could also contribute to these effects. Indeed, further studies are necessary to confirm these results and fully understand each olive oil type’s specific impacts [60].

As part of the Prevención con Dieta Mediterránea (PREDIMED) nutrition intervention trial, 447 cognitively healthy volunteers with high cardiovascular risk were assessed to understand the impact of a Mediterranean diet supplemented with antioxidant-rich foods on cognitive function. The participants were divided into three groups according to the diets they followed: a Mediterranean diet supplemented with olive oil, a Mediterranean diet with mixed nuts, and a control diet with advice to reduce dietary fat. After a median follow-up of 4.1 years, the results indicated that the groups who followed the Mediterranean diet supplemented with olive oil or nuts exhibited improved cognitive function compared to the control group (control diet with advice to reduce dietary fat), with particular improvements noted in memory and frontal cognition tests. The findings from this study provided compelling evidence that a Mediterranean diet supplemented with olive oil can play a crucial role in improving cognitive function, particularly in memory and frontal cognition [93].

Another pivotal clinical trial within the PREDIMED study evaluated 522 high-vascular-risk participants, with an average age of 74.6 years, over 6.5 years [94]. Similarly, this study focused on the cognitive effects of the Mediterranean diet supplemented with olive oil or nuts compared to a low-fat control diet. Consistently, the findings revealed significant improvements in cognitive performance, as measured by the MMSE and the Clock Drawing tests, in participants who consumed the Mediterranean diet enhanced with EVOO or nuts, highlighting the diet’s effectiveness in boosting cognitive health in older individuals at high vascular risk [94].

Furthermore, a randomized cross-over clinical trial investigated the combination of OLE and S-acetyl glutathione on cognitive and behavioral functions in 18 patients with mild AD [95]. Despite challenges posed by the COVID-19 pandemic, significant improvements in cognitive function were observed. Notable enhancements were seen in cognitive deterioration, memory, visuospatial abilities, attention, language, executive functions, and behavioral disorders, emphasizing the potential efficacy of dietary supplementation with olive polyphenols and bioavailable glutathione in mild AD patients [95]. The findings from this study support that dietary supplementation with OLE and S-acetyl glutathione can significantly improve cognitive and behavioral functions in mild AD patients.

**Figure 3:**
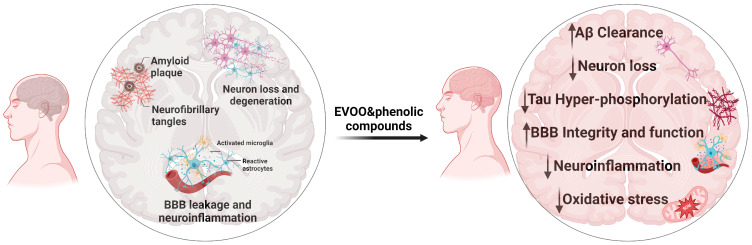
Neuropathological key features of AD (**left**) contrasted with the neuroprotective effects of EVOO and phenolic compounds (**right**).

Collectively, the findings from in vitro, preclinical in vivo, and human studies demonstrated that EVOO and its phenolic compounds could be protective against AD. A summary of EVOO and its phenolics’ beneficial effects against AD is shown in Figure 3.

**Table 2:** Summary of completed and ongoing clinical studies evaluating the effects of olive oil and derivatives on cognitive functions.

Conducted Clinical Studies			
Trial	Intervention	Findings	Citation
The Italian Longitudinal Study on Aging (ILSA)	Mediterranean diet including olive oil	Inverse relationship between MUFA intake and cognitive decline in subjects with MMSE scores < 24	[88]
The Prevención con Dieta Mediterránea (PREDIMED)	Mediterranean diet supplemented with olive oil, or Mediterranean diet with mixed nuts, or control diet. Subjects were followed up for 4.1 years on average	The Mediterranean diet supplemented with olive oil or nuts group had improved cognitive function compared to the control group and improved performance in the MMSE and the Clock Drawing tests	[93,94]
The MICOIL pilot study	Greek high-phenolic early-harvest EVOO (HP-EH-EVOO), or moderate-phenolic EVOO (MP-EVOO), or Mediterranean diet in individuals with MCI	- HP-EH-EVOO exhibited better cognitive performance than the MP-EVOO and Mediterranean diet groups, regardless of the APOEɛ4 genotype. HP-EH-EVOO also enhanced brain connectivity and functionality - MP-EVOO showed significant improvement in specific cognitive tests compared to the Mediterranean diet group	[91,92]
The AU-ROOAD pilot study	EVOO or ROO in individuals with MCI	- ROO and EVOO improved CDR and behavioral scores - EVOO and ROO reduced blood biomarkers related to AD pathology - EVOO reduced BBB permeability and enhanced functional connectivity in the brain - ROO increased functional brain activation in a memory task	[60]
Marianetti et al. (2022)	OLE and S-acetyl glutathione dietary supplement in individuals with AD	OLE and S-acetyl glutathione improved memory, visuospatial abilities, attention, language, executive functions, and behavioral disorders	[95]
**Ongoing pilot trials**	**Objective**		
Management of Dementia with Olive Oil Leaves—GOLDEN (NCT04440020)	Evaluate and compare olive-leaf beverage and Mediterranean diet effects on memory and cognitive function in individuals with mild dementia		
Pilot Study About Extra Virgin Olive Oil “Coratina” in Mild Cognitive Impairment and Alzheimer’s Disease Patients—EVOCAD (NCT04229186)	Evaluate and compare EVOO and ROO effects on cognitive and heart functions in individuals with MCI and AD		
Does EVOO Induce Gene and Metabolic Changes in Healthy Subjects? (NCT05929924)	Evaluate EVOO effect on molecular and biological pathways linked with AD in healthy subjects with AD family history		

## 6. Conclusions

In conclusion, the significance of EVOO and its phenolic compounds in the context of AD is pronounced, especially considering that AD is a condition that begins years before symptoms appear, underscoring the critical need for preventive approaches in the face of the current lack of curative treatments. EVOO and olive phenolics potentially mitigate critical pathological features of AD, such as Aβ and tau protein pathologies, oxidative stress, and neuroinflammation, and enhance BBB integrity. Given the complexities and limitations of existing AD medications, further comprehensive clinical trials are crucial to validate the efficacy and safety of EVOO and its phenolic compounds. This review advocates incorporating EVOO and its derivatives into diets as a proactive measure for neurological health, paving the way for future research and strategies in effective AD prevention and management.

The promising outcomes of pilot studies underline the urgency of further research into EVOO as a medical food and warrant its consideration in dietary guidelines for those at risk of neurodegenerative diseases. Our advocacy for the inclusion of EVOO and its derivatives in the diet is based on a growing body of evidence suggesting a shift towards more effective strategies against the complex challenge of AD, emphasizing not just treatment but prevention and early intervention.

Despite all the evidence presented in this review that has delineated the beneficial effect of EVOO, there remains a crucial need for future research to solidify these findings and guide clinical applications. Prospective studies should focus on large-scale, longitudinal, multicentric clinical trials to definitively establish the therapeutic efficacy and safety of EVOO and its phenolic compounds. Such research should aim to elucidate the molecular mechanisms underlying the observed neuroprotective effects, which could pave the way for developing novel therapeutics based on these natural compounds. Further exploration into the synergistic effects of EVOO phenolic compounds with existing AD treatments may unveil comprehensive management strategies, potentially leading to integrated therapeutic approaches. Additionally, the preventive potential of EVOO in at-risk populations deserves further exploration, as does the role of dietary patterns and the timing of EVOO consumption in AD prevention. Future investigations should also consider genetic factors, like the APOEɛ4 allele, to tailor dietary interventions.

## Figures and Tables

**Table 1:** Summary of in vitro and in vivo studies evaluating the effects of olive oil and derivatives in preclinical models.

**In Vitro Studies**			
Cell Line	Intervention	Findings	Reference
SH-SY5Y	EVOO (10 µg/mL)	- Upregulated antioxidant enzymes	[63]
	OLG and HT (75 μM)	- Activated autophagic flux - Reduced cell damage, ROS production, and mitochondrial impairment	[62]
	OC and ligstroside (0.05 µM)	- Increased basal ATP levels - Enhanced respiratory chain complexes - Enhanced mitochondrial biogenesis genes	[70]
	Olive leaf extract (20–40 μg/mL)	- Increased specific phospholipids - Protected against Aβ_42_-induced cell death - Decreased triacylglycerols	[77]
	HT-B (5 and 10 μM) for 6 h, then 6-OHDA (100 μM) for 18 h	- Inhibited apoptosis - Induced Nrf2 and HO-1 - Reduced oxidative damage and apoptosis	[78]
	OLG and HT, 2:1 (50 μM:25 μM), 1:1 (37.5 μM:37.5 μM), and 1:2 (25 μM:50 μM	- Prevented the formation and growth of toxic Aβ42 oligomers - Accelerated the formation of non-toxic fibrils - Stabilized Aβ42 oligomers and fibrils - Reduced neurotoxic effects	[76]
RAW 264.7	HT (200 µM and 50 µM)	- Inhibited iNOS and COX-2 expression - Reduced cytokine and chemokine secretion via the NF-κB pathway	[65,66,67,68]
bEnd3	OC (0.5–50 µM)	- Increased expression and function of P-gp and LRP1 - Enhanced Aβ degradation	[74,75]
CCF-STTG1	OC (5 µM)	- Mitigated Aβo-induced synaptic protein - Reduced inflammation - Preserved neuro-supportive functions	[31]
7PA2	HT (5 µM)	- Corrected mitochondrial energetic dysfunction - Increased ATP levels - Enhanced mitochondrial number and fusion	[79]
BV2	MVOO-PE and EVOO extract (1, 2, 5, 10, and 20 µg/mL)	- Reduced cell death - Attenuated TLR4/NLRP3 pathway - Decreased inflammatory markers	[80]
**In Vivo Studies**			
Model	Intervention	Results	Reference
TgSwDI	EVOO (0.7 g/kg/day) daily for 3–6 months	– Reduced total Aβ with 3–6 months of treatment and tau levels after 6 months of treatment – Enhanced clearance of Aβ	[52]
3xTg	EVOO-enriched diet for 3 and 6 months (dose not described)	– Enhanced behavioral performance – Increased synaptic integrityReduced brain Aβ levels – Decreased tau phosphorylation – Increased autophagy	[81]
TgCRND8	OLG (50mg/kg) for 8 weeks	– Reduced Aβ plaque load – Enhanced autophagy – Restored lysosomal system	[82]
5xFAD	Oleuropein-rich olive oil (695 μg/kg/day) for 3 months	– Reduced Aβ brain load – Increased Aβ clearance and induction of nonamyloidogenic pathway – Reduced neuroinflammation – Diminished expression of RAGE and HMGB1 – Enhanced synaptic markersImproved memory performance	[83]
TgSwDI	OC-rich EVOO daily for three months (OC at 476 µg/Kg/day)	– OC-rich EVOO restored BBB function – Reduced neuroinflammation – Reduced brain Aβ load – Prompted autophagy	[59]
5xFAD	OC 10mg/kg for 3 months	– Improved altered metabolic and behavior parameters – Alleviated anxiety-like behavior and sleep disturbances	[84]
NMRI	OC or ligstroside for 6.25 mg/kg 6 months	– Improved spatial working memory – Restored brain ATP levels – Extended lifespan	[70]
5xFAD	OC (0.5 mg OC/kg/day) or EVOO low in OC (0.5 mg total phenols/kg per day) for 3 months	– Both OC and OC-low EVOO reduced brain Aβ levels and neuroinflammation – Both OC-low EVOO and OC improved the integrity of the BBB – Both reduced brain total Aβ load by increasing clearance and reducing production – OC alone suppressed RAGE/HMGB1 pathway	[57]
TgCRND8	OLE (50 mg/kg of diet) in low-fat (5.0%) AIN-76A diet for 8 weeks	– Autophagy activation mediated by mTOR inhibition	[85]
TgCRND8	HT (50 mg/kg of diet) with low-fat diet for 8 weeks	– Reduction in neuroinflammation – Reduction in brain Aβ load – Induction of macroautophagic – Improved memory	[87]
APP/PS1	HT (5 mg/kg/day) for 6 months	– Modulated mitochondrial oxidative dysfunction – Reduced brain inflammation	[86]
5xFAD	EVOO (0.7 g/kg/day for 3 months) with or without donepezil (1 mg/kg/day for 1 month)	– EVOO enhanced the noncholinergic mechanisms of donepezil by reducing brain Aβ load – Combination increased Aβ clearance pathway and reduced production – Upregulated synaptic protein expression – Decreased neuroinflammation	[31]
